# Author Correction: All-optical switching of an epsilon-near-zero plasmon resonance in indium tin oxide

**DOI:** 10.1038/s41467-021-22020-7

**Published:** 2021-03-04

**Authors:** Justus Bohn, Ting Shan Luk, Craig Tollerton, Sam W. Hutchings, Igal Brener, Simon Horsley, William L. Barnes, Euan Hendry

**Affiliations:** 1 School of Physics, University of Exeter, Exeter, UK; 2 Sandia National Laboratories, Albuquerque, NM USA; 3 Center for Integrated Nanotechnologies, Sandia National Laboratories, Albuquerque, NM USA

**Keywords:** Nanophotonics and plasmonics, Nonlinear optics, Sub-wavelength optics, Ultrafast photonics

Correction to: *Nature Communications* 10.1038/s41467-021-21332-y, published online 15 February 2021.

The original version of the Supplementary Information associated with this Article contained an error in Supplementary Figure 5, in which panel c of Supplementary Figure 5 had an error on the vertical axis. The HTML has been updated to include a corrected version of the Supplementary Information.

## Supplementary information

Supplementary material

