# Supplementary material for: All-optical switching of an epsilon-near-zero plasmon resonance in indium tin oxide
Source: Nat Commun. 2021 Feb 15;12:1017. doi: 10.1038/s41467-021-21332-y (PMC7884677; doi:10.1038/s41467-021-21332-y)
Supplement: Supplementary file 1 — Supplementary Information [file 41467_2021_21332_MOESM1_ESM.pdf]

Supplementary material: All-Optical Switching of an Epsilon-Near-Zero Plasmon Resonance in Indium Tin Oxide

Justus Bohn[1,*], Ting Shan Luk[2,3], Craig Tollerton[1], Sam Hutchins[1], Igal Brener[2,3], Simon Horsley[1], William L. Barnes[1], and Euan Hendry[1]

[1]School of Physics, University of Exeter, EX4 4QL, UK
[2]Sandia National Laboratories, Albuquerque, 87185, New Mexico, USA
[3]Center for Integrated Nanotechnologies, Sandia National Laboratories, Albuquerque, 87185, New Mexico, USA
[*]jb933@exeter.ac.uk

Contents

S1 ITO permittivity (Drude model) **1**
S2 Three layer dispersion **1**
S3 Model: Intensity dependent reflection **1**
S3.1 Angle and frequency dependent reflection (Static model): 2
S3.2 Time varying medium (Dynamic model) 3
S4 Two-beam coupling contribution **6**
S5 Probe polarization dependence of the nonlinear effect **7**
S6 TE-TE measurement **8**
S7 Coverslip measurement **9**
S8 Autocorrelation measurement **9**
References **9**

S1 ITO permittivity (Drude model)

The near-infrared permittivity of the ITO film was measured with an ellipsometer and can be described by the Drude model:

$$\varepsilon_{\mathrm{ITO}}(\omega) = \varepsilon_\infty - \frac{\omega_\mathrm{p}^2}{\omega^2 + i\omega\gamma} \tag{S1}$$

with $\varepsilon_\infty = 3.43$ as the high-frequency permittivity, $\omega_\mathrm{p} = 2.86 \times 10^{15}$ rad/s as the bulk plasmon frequency and $\gamma = 2.24 \times 10^{14}$ rad/s as the damping rate. These parameters were determined experimentally by ellipsometry and subsequent fitting.

S2 Three layer dispersion

The dispersion of ENZ plasmon modes can be found using the dispersion relation taken from Ref.[1]:

$$F(k,\omega) = \tanh(\alpha_2 d) + \frac{\varepsilon_2\alpha_2(\varepsilon_1\alpha_3 + \varepsilon_3\alpha_1)}{\alpha_2^2\varepsilon_1\varepsilon_3 + \alpha_1\alpha_3\varepsilon_2^2} = 0, \tag{S2}$$

with $\alpha_j^2 = k^2 - k_0^2\varepsilon_j$. The permittivities are $n_1 = 1$ (air), $\varepsilon_2 = \varepsilon_{\mathrm{ITO}}(\omega)$ and $n_3 = 1.43$ (substrate). We solve for a complex wavevector $k = k_r + ik_i$ and real frequency ω.

S3 Model: Intensity dependent reflection

We introduce two models that we use to interpret our pump-probe data. The first "static" model treats our sample as a homogeneous layer, and we solve for excited state permittivity parameters. The second "dynamic" model predicts the full dynamic behaviour of samples assuming a time dependent plasmon resonance frequency, and allows us to predict the temporal dynamics observed in our pump-probe measurements.

S3.1 Angle and frequency dependent reflection (Static model):

We use a transfer matrix approach to calculate the reflection and absorption of a beam from our sample, assuming a given permittivity. The initial absorption is calculated for the TE polarized pump beam $A_{\mathrm{pm}}\,(\theta_{\mathrm{pm}}, f_{\mathrm{pm}})$, which can be seen in Figure S1. The incoming medium is assumed to be substrate/prism (index n = 1.43), while the medium on the far side is air (n=1). The

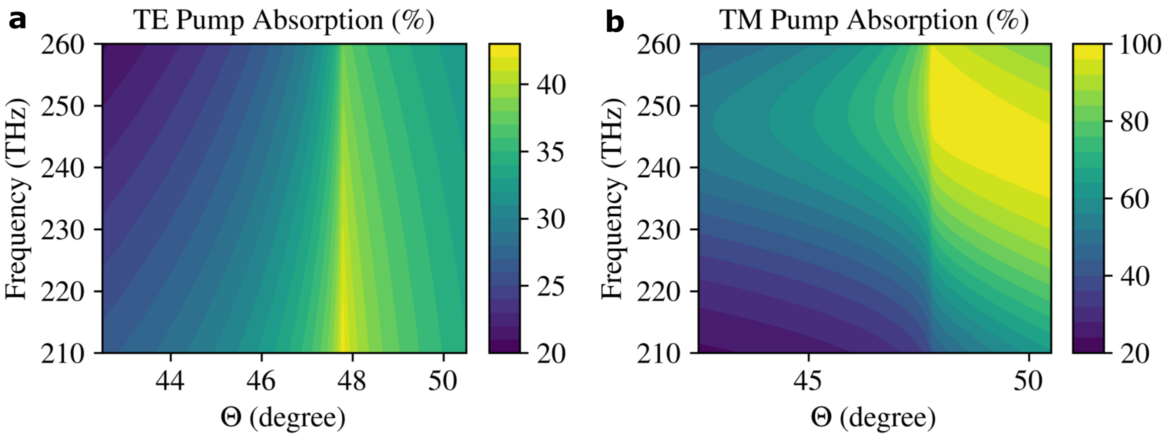

Figure S1. Pump absorption. a, The calculated TE pump absorption based on ellipsometric data. Due to $\theta = \theta_{\mathrm{pr}} = \theta_{\mathrm{pm}} + 3.4^{\circ}$ we find the largest absorption close to 48°, the critical angle of the pump beam. **b**, The calculated TM pump absorption, which provides a strong absorption through the resonant ENZ plasmon.

calculated $A_{\mathrm{pm}}\,(\theta_{\mathrm{pm}}, f_{\mathrm{pm}})$ is used to determine the angle and frequency dependent absorbed pump intensity

$$I = \cos\left(\theta_{\mathrm{pm}}\right) A_{\mathrm{pm}}\left(\theta_{\mathrm{pm}}, f_{\mathrm{pm}}\right) I_{\mathrm{external}}. \qquad \text{(S3)}$$

The intensity dependent ITO is modelled as an effective medium layer. We assume a linear intensity dependence of the Drude permittivitty parameters, such that the plasma frequency can be written as:

$$\omega_{\mathrm{p}}(I) = (1 + \omega_{\mathrm{p},2} I)\,\omega_{\mathrm{p},0}. \qquad \text{(S4)}$$

We use this to calculate the pump induced reflection of the probe $R_{\mathrm{pumped}}(\theta_{\mathrm{pr}}, f_{\mathrm{pr}}, I)$. By least square fitting of the proportionality factor, $\omega_{\mathrm{p},2}$, to the experimental data in Fig. 2c of the main manuscript, we find $\omega_{\mathrm{p},2} = -0.38\ \%\,\mathrm{cm}^2\,\mathrm{GW}^{-1}$. The decrease in ω_{p} with increasing pump intensity can be understood as an increase of effective mass upon exciting the electrons in the non-parabolic conduction band of ITO, as discussed in refs[2–5].

Several references in the literature have also reported simultaneous intensity dependent changes to the scattering rate γ[2,3]. However, using our full dynamic model (introduced below), the most convincing reproduction of our measured pump-probe dynamics is found when γ is independent or weakly dependent ($\gamma_2 <= 0.5\,\%\,\mathrm{cm}^2\,\mathrm{GW}^{-1}$) on intensity. We see this by comparing the experimental measurements (Figure 2b and Figure S2a) with the cases of $\gamma_2 = 0$ (Figure S2b), $\gamma_2 = 5\,\%\,\mathrm{cm}^2\,\mathrm{GW}^{-1}$ (Figure S2c), $\gamma_2 = 0.5\,\%\,\mathrm{cm}^2\,\mathrm{GW}^{-1}$ (Figure S2d) and $\gamma_2 = -0.5\,\%\,\mathrm{cm}^2\,\mathrm{GW}^{-1}$ (Figure S2e). Moreover, fitting our angle and frequency dependent results assuming both $\omega_{\mathrm{p},2}$ and a similarly defined γ_2 leads to an unphysical effect: a predicted small decrease in γ with increasing intensity, which arises due to a narrowing of the resonance feature observed in the R_{pumped} data in Fig. 3 for increasing intensity. This is an artefact of our data analysis, due to the oscillatory features for case II (where the ENZ resonance shifts spectrally through the probe) which become more prominent for increasing intensity. In any case, our results suggest that the nonlinear switching we observe in our samples is primarily due to intensity dependent changes to ω_{p}, while intensity dependent changes to γ are not clearly identifiable in our experimental data.

Finally, we can contrast the magnitude of the observed optical nonlinearity with the most comparable data previously reported in the literature. Our fit result $\omega_{\mathrm{p},2} = -0.38\,\%\,\mathrm{cm}^2\,\mathrm{GW}^{-1}$ is surprisingly similar to that report by Alam et al.[2], corresponding to $\omega_{\mathrm{p},2} = -0.33\,\%\,\mathrm{cm}^2\,\mathrm{GW}^{-1}$, found from the absorbed intensity dependence plotted in Figure S3a. However, this is unexpected, as the nonlinear response should be dictated by absorbed energy density, and the sample thickness is different by a factor of ~ 5 (310 nm, while here 60 nm). In Figure S3b we plot the calculated absorption per length using transfer matrix modelling[6] as an indicator of absorbed energy density, which eventually causes the electron heating[5]. For our Kretschmann-Raether geometry with a film thickness of 60 nm, the absorbed energy density is reasonably constant across the layer (see blue line in figure Figure S3b). However, for direct transmission from air with a 310 nm thick film, the geometry

used in ref[2], a significant exponential decay of energy density is expected across the film, with a decay length of 168 nm. This means that the Drude parameters in this case are not expected to be homogeneous, but will be strongly dependent on spatial position in the sample. Nevertheless, as a guide one would expect $\omega_{p,2}$ to be lower, by around a factor of 3, for the geometry used in ref[2] compared to the Kretschman-Raether geometry used here. We do not fully understand this discrepancy, but it may arise due to complications in the homogeneous analysis used in ref[2], or due to variations between ITO samples.

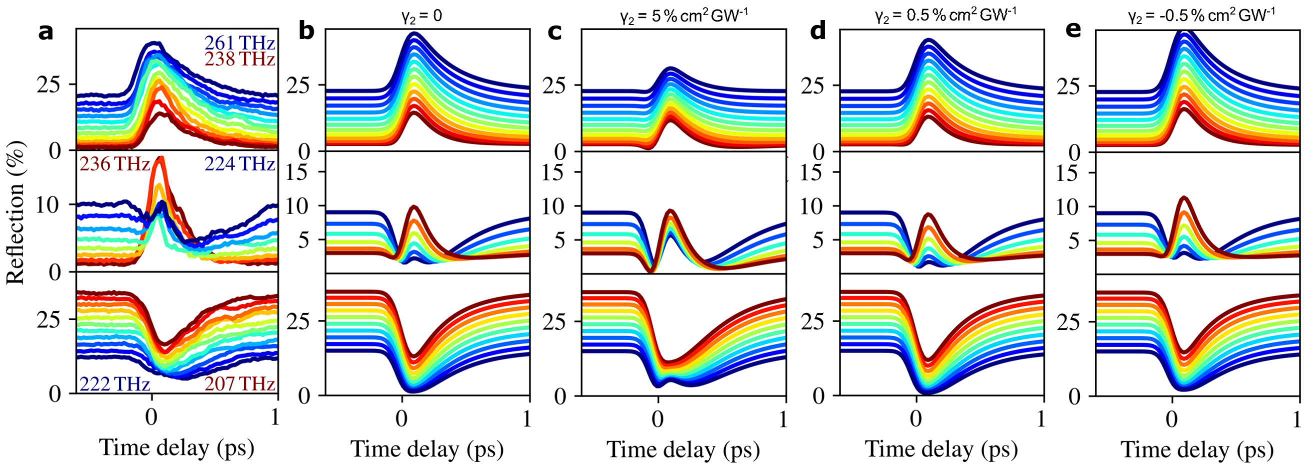

Figure S2. Impact of γ_2 on model. **a**, Time delay scans presented in Fig. 2b of the manuscript (pr: TM, 48.3°, $f_{\mathrm{pr}} = 261\ldots207$ THz; pm: TE, 44.9°, $f_{\mathrm{pm}} = f_{\mathrm{pr}}$). **b**, Dynamic model results for $\omega_{p,2} = -0.38\,\%\,\mathrm{cm^2\,GW^{-1}}$, estimated by fitting Fig. 2c with an intensity dependent ω_p only. **c**, Adding a $\gamma_2 = 5\,\%\,\mathrm{cm^2\,GW^{-1}}$, increases the estimated pumped resonance width. This leads to a reduced absorption depth of the switched plasmon (reflection doesn't go to zero), which is in line with experiments, but also leads to a decreased nonlinear reflection for the high frequencies, which is in this size incompatible with our findings. **d**, A more moderate effect given by up to $\gamma_2 \sim 0.5\,\%\,\mathrm{cm^2\,GW^{-1}}$ could still be in-line with our measurements. **e**, $\gamma_2 = -0.5\,\%\,\mathrm{cm^2\,GW^{-1}}$, has the opposite effect by even further increasing the nonlinear reflection for the high frequency case. Moreover, a scattering rate which decreases with increasing electron temperature seems unphysical.

Refractive index

To enable comparison to other studies using the refractive index and n_2 as the nonlinear optical parameters we provide the nonlinear refractive index depending on the absorbed intensity:

$$n(I_{\mathrm{abs}}) = n_0 + n_2 I_{\mathrm{abs}} + O(I_{\mathrm{abs}}^2) \tag{S5}$$

with

$$n_0 = \sqrt{\varepsilon_\infty - \frac{\omega_{p,0}^2}{\omega^2 + i\omega\gamma_0}} \tag{S6}$$

$$n_2 = \frac{\omega_{p,0}^2}{2n_0}\frac{2\omega^2\omega_{p,2} - i\omega\gamma_0(\gamma_2 - 2\omega_{p,2})}{(\omega^2 - i\omega\gamma_0)^2} \tag{S7}$$

To give an example, n_2 takes the value of $(0.01 - i * 0.016)\,\mathrm{cm^2\,GW^{-1}}$ for our ITO sample case and 1250 nm wavelength. This equation may result in an approximate conversion for other ITO sample designs, but $\omega_{p,2} = 0.38\,\%\,\mathrm{cm^2\,GW^{-1}}$ is expected to be different for materials with different band-structures such as AZO. Furthermore, to compare between different layer thicknesses and/or pulse lengths, one should also consider that the absorbed energy density is the expected scaling parameter of the thermal nonlinearity, not the absorbed intensity.

S3.2 Time varying medium (Dynamic model)

We now concentrate on modelling the full temporal dynamics of a probe pulse experiencing a time dependent modulation of medium's dielectric function, induced by the pump pulse. Without any time modulation of the permittivity, Maxwell's equations for an incident TM polarized wave are

$$\nabla \times \mathbf{E} = -\mu_0 \frac{\partial H}{\partial t}\hat{\mathbf{z}} \tag{S8}$$

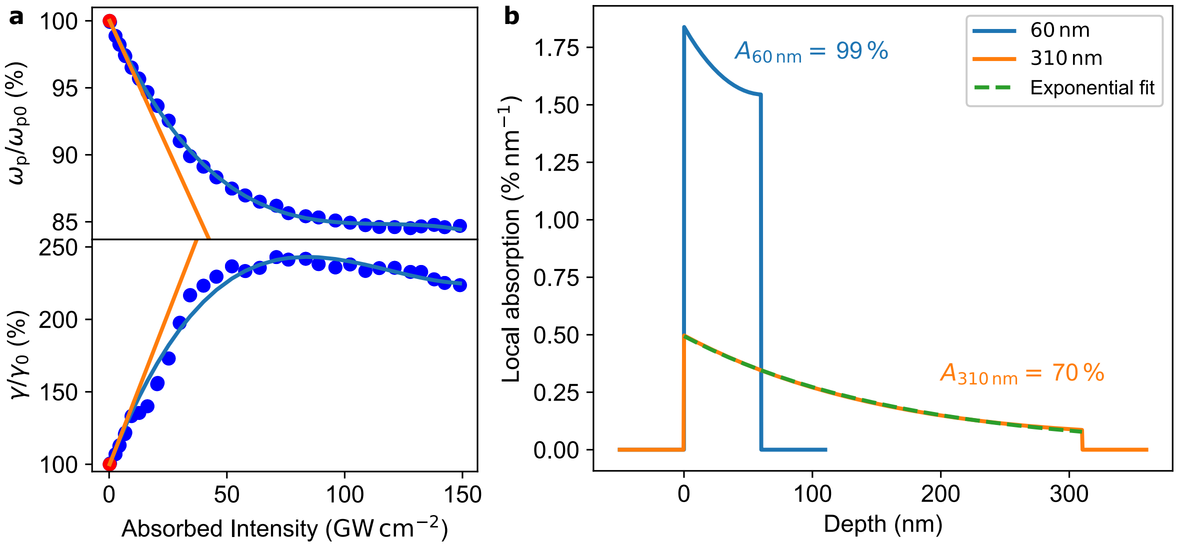

Figure S3. Comparison of different sample thickness. We compare to the previous studies studies of Alam et al.[2] which features a 310 nm ITO layer with similar optical properties to ours (TM polarized). **a**, We convert the given intensity dependent complex refractive index given in[2] to ω_p and γ by assuming a Drude model. Linear contributions for the low intensity fit region give $\omega_{p,2} = -0.33\,\%\,\text{cm}^2\,\text{GW}^{-1}$ and $\gamma_2 = 4.6\,\%\,\text{cm}^2\,\text{GW}^{-1}$. **b**, The 310 nm film (in air, $\theta = 30°$, $\lambda = 1240$ nm) produces an absorbed energy density which varies with distance, and is described by an exponential decay length of $\tau = 168$ nm. While the 60 nm film (prism, $\theta = 45°$, $\lambda = 1200$ nm) exhibits a relatively constant absorption throughout the layer.

and

$$\nabla H \times \hat{\mathbf{z}} = \frac{\partial \mathbf{D}}{\partial t} = \varepsilon_0 \frac{\partial}{\partial t}\left[\mathbf{E}(t) + \int_0^\infty \chi\left(\frac{t'}{\Delta}\right)\mathbf{E}\left(t-t'\right)dt'\right] \tag{S9}$$

where $\chi\left(\frac{t'}{\Delta}\right)$ is the time domain susceptibility, and Δ is the characteristic timescale of the material response. In our case the susceptibility function χ itself varies in time due to the heating induced changes of the plasmon frequency ω_p (see Figure S4). The later are modelled as a convolution of the pump pulse and an exponential decay with $\tau = 300$ fs, normed such that ω_p given in Equation S4 corresponds to the extremum. We thus modify (S9) so that the susceptibility has an additional dependence on $t - t'$,

$$\nabla H \times \hat{\mathbf{z}} = \varepsilon_0 \frac{\partial}{\partial t}\left[\mathbf{E}(t) + \int_0^\infty \chi\left(\frac{t-t'}{\Delta_1}, \frac{t'}{\Delta_2}\right)\mathbf{E}\left(t-t'\right)dt'\right] \tag{S10}$$

We now have two timescales Δ_1 (the modulation of the permittivity), and Δ_2 (the timescale over which the material responds). These timescales are assumed to be such that the modulation is much slower than the material response $\Delta_1 \gg \Delta_2$ and the material response tends to zero at large delay times $\chi(\tau_1, \tau_2 \to \infty) = 0$. Taking the curl of (S10) and applying (S8) we find the equation for the out of plane magnetic field in a homogeneous region of space

$$\nabla^2 H - \frac{1}{c^2}\frac{\partial^2 H}{\partial t^2} - \frac{1}{c^2}\frac{\partial}{\partial t}\int_0^\infty \chi\left(\frac{t-t'}{\Delta_1}, \frac{t'}{\Delta_2}\right)\frac{\partial H\left(t-t'\right)}{\partial t}dt' = 0 \tag{S11}$$

In general this is an integro-differential equation that is difficult to solve. To make progress we use multiple scales perturbation theory[7], and define two time variables $\tau_1 = t/\Delta_1$ and $\tau_2 = t/\Delta_2$. The out of plane magnetic field is approximated as a function of these two time variables

$$H(t) = H\left(\frac{t}{\Delta_1}, \frac{t}{\Delta_2}\right) = H\left(\tau_1, \tau_2\right) \tag{S12}$$

and the time derivatives can be written as

$$\frac{\partial}{\partial t} = \frac{1}{\Delta_1}\frac{\partial}{\partial \tau_1} + \frac{1}{\Delta_2}\frac{\partial}{\partial \tau_2} \tag{S13}$$

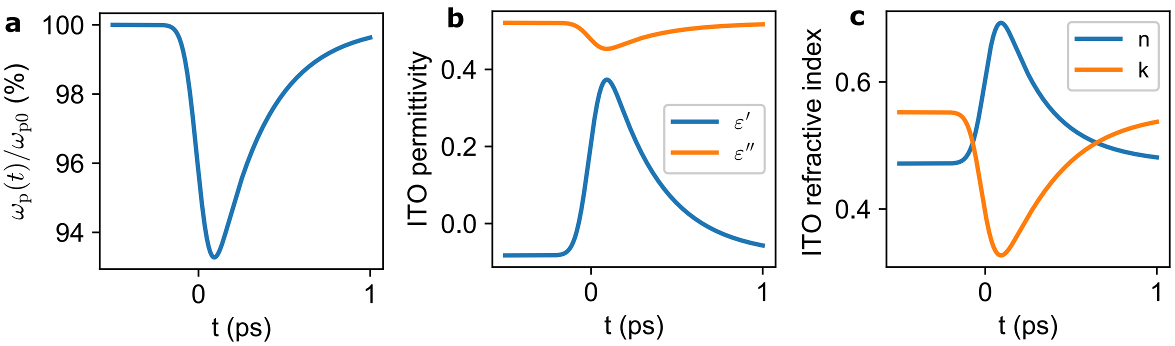

Figure S4. Time dependent bulk plasmon frequency. The convolution of the pump pulse and an exponential decay with $\tau = 300$ fs is normed such that $\omega_p(I)$, given in Equation S4, corresponds to the extremum. **a**, The $\omega_p(t,I)$ is used to calculate the time dependent material parameters such as χ during interaction with the pump beam. For the plotted case we assume $\theta = 48.3°$, $I_{\text{external}} = 70\,\text{GW}\,\text{cm}^{-2}$ and a TE polarized pump (as seen in Fig. 2b). For the case of $f_{\text{pr}} = 240$ THz we plot the corresponding permittivity (**b**) and refractive index (**c**).

Given that $\chi(\tau_1 - \tau_1', \tau_2')$ decays with increasing τ_2' over which time τ_1' has not increased appreciably from zero we can expand the integral kernel around $\tau_1' = 0$ with the leading order approximation being $\chi(\tau_1 - \tau_1', \tau_2') \sim \chi(\tau_1, \tau_2')$. Further terms are proportional to increasing powers of Δ_2/Δ_1. Equation (S11) is thus to leading order in the small quantity Δ_2/Δ_1

$$\nabla^2 H(\tau_1, \tau_2) - \frac{1}{\Delta_2^2 c^2} \frac{\partial^2 H(\tau_1, \tau_2)}{\partial \tau_2^2} - \frac{1}{\Delta_2^2 c^2} \int_0^\infty \chi(\tau_1, \tau_2') \frac{\partial^2 H(\tau_1, \tau_2 - \tau_2')}{\partial \tau_2^2} d\tau_2' = 0 \tag{S14}$$

Equation (S14) is different from (S11) in an important way. Having dropped the terms involving the timescale $\tau_1 = t/\Delta_1$ from the integral and the derivatives, the modulation of the dielectric function neither appears in the derivatives nor the integration. The time variable τ_1 is now merely a label, and we may solve (S14) as we would in the time independent situation, with the dispersive permittivity $\varepsilon(\tau_1, \omega)$. Note that to improve upon this approximation, one can add back in the terms proportional to the ratio of timescales Δ_2/Δ_1, expanding perturbatively.

We now apply this result to the experiment. We have a total internal reflection geometry with fixed permittivities, $\varepsilon_1 = 2.1$ in the region $x < 0$, and $\varepsilon_3 = 1$ in the region $x > d$. In the remaining region $0 < x < d$ we have a layer of material with a permittivity that is modulated over time $\varepsilon_2(\tau_1, \omega)$ with the same separation of timescales assumed above. A pulse is incident at a fixed angle θ from the region $x < 0$. The reflection coefficient (which, as with the permittivity is now a function of modulation time τ_1, and incidence frequency ω) is given by

$$r(\tau_1, \omega) = \frac{\cos(k_2 d)\left(\frac{k_2}{k_3 \varepsilon_2} - \frac{k_2 \varepsilon_1}{k_1 \varepsilon_2}\right) - i \sin(k_2 d)\left(1 - \frac{k_2^2 \varepsilon_1}{k_1 k_3 \varepsilon_2^2}\right)}{\cos(k_2 d)\left(\frac{k_2 \varepsilon_1}{k_1 \varepsilon_2} + \frac{k_2}{k_3 \varepsilon_2}\right) - i \sin(k_2 d)\left(1 + \frac{k_2^2 \varepsilon_1}{k_1 k_3 \varepsilon_2^2}\right)}, \tag{S15}$$

where $k_0 = \omega/c$, $k_1 = k_0\sqrt{\varepsilon_1}\cos(\theta)$, $k_2 = k_0\sqrt{\varepsilon_2(\tau_1, \omega) - \varepsilon_1 \sin^2(\theta)}$, and $k_3 = k_0\sqrt{1 - \varepsilon_1 \sin^2(\theta)}$. If the spectrum of the incident pulse is $a(\omega)$ then the total pulse in the first region $x < 0$ is the sum of the incident spectrum plus the incident spectrum weighted by the modulated reflection coefficient $r(\tau_1, \omega)$:

$$\begin{aligned} H(\tau_1, \tau_2) = \quad & H_{\text{inc}}(\tau_1, \tau_2) + H_r(\tau_1, \tau_2) \\ = & \int_{-\infty}^{\infty} a(\omega) \left[e^{ik_0(\cos(\theta)x - c\Delta_2 \tau_2)} + r(\tau_1, \omega) e^{-ik_0(\cos(\theta)x + c\Delta_2 \tau_2)}\right] \frac{d\omega}{2\pi} \\ = & \int_{-\infty}^{\infty} a(\omega) \left[e^{ik_0(\cos(\theta)x - ct)} + r\left(\frac{t}{\Delta_1}, \omega\right) e^{-ik_0(\cos(\theta)x + ct)}\right] \frac{d\omega}{2\pi}. \end{aligned} \tag{S16}$$

The spectrum of the reflected pulse is found by simply taking the Fourier transform of the second term in the square brackets of (S16), at $x = 0$:

$$H_r(\omega) = \int_{-\infty}^{\infty} H_r(t) e^{i\omega t} dt = \int_{-\infty}^{\infty} \frac{d\omega'}{2\pi} a(\omega') r(\omega - \omega', \omega'), \tag{S17}$$

where

$$r(\omega_1, \omega_2) = \int_{-\infty}^{\infty} r\left(\frac{t}{\Delta_1}, \omega_2\right) e^{i\omega_1 t} dt. \tag{S18}$$

Equation (S17) indicates that the spectrum of the reflected pulse is the convolution of the incident spectrum with the two frequency reflection coefficient $r(\omega_1, \omega_2)$.

Finally, to compare to experiment, we calculate the relative reflected power given in the main text. To do this we then take ratio of the integrated incident and reflected Poynting vectors. For instance, the time integrated incident power is

$$P_{\text{inc}} = \int_{-\infty}^{\infty} \hat{x} \cdot S(t) dt = \int_{-\infty}^{\infty} E_{y,\text{inc}}(t) H_{\text{inc}}(t) dt = \frac{\cos(\theta)}{c\varepsilon_0 \sqrt{\varepsilon_1}} \int_{-\infty}^{\infty} \frac{d\omega}{2\pi} |a(\omega)|^2, \tag{S19}$$

and therefore the relative reflected power is

$$R = \frac{\int_{-\infty}^{\infty} \frac{d\omega}{2\pi} |H_r(\omega)|^2}{\int_{-\infty}^{\infty} \frac{d\omega}{2\pi} |a(\omega)|^2}, \tag{S20}$$

where $H_r(\omega)$ is calculated using Eq. (13), and the time-frequency reflection coefficient in Eq. (S15). Equation (S20) is used to calculate the reflected power for the different pump pulse arrival times in Fig. 2b.

The results of our model are shown in Fig. 2b, with time dynamics split into three distinct cases. Case I: the ENZ resonance red shifts away from the probe, the absorption decreases and we see an increase in reflectivity due to the pump excitation. Case III: the ENZ resonance shifts spectrally towards the probe, the absorption increases and we see a decrease in reflectivity. Both cases display expected temporal dynamics: a fast, $\sim$100 fs (pulse limited) initial change, followed by a slower , $\sim$1 ps thermal relaxation, similar to that observed in experiment here and in previous studies[2,8]. We use the magnitudes of reflection changes in these regions to find our intensity dependent Drude parameters, $\omega_{\text{p},2}$ and γ_2. We find best agreement with the data for $\omega_{\text{p},2} = -0.38\,\%\,\text{cm}^2\,\text{GW}^{-1}$. Including a non-zero γ_2 leads to a decreased nonlinear reflection for either case I or III, depending on sign. Both cases, i.e. γ significantly increasing or decreasing with intensity, therefore lead to discrepancies with the experimental data for regions I or III.

Finally, we have case II: the ENZ resonance shifts spectrally through the probe, for which we observe some rather unusual dynamics in the pump-probe signal. While we are able to reproduce similar oscillatory features with our model, these features are smaller in the model when compared to experiment. This may be due to a very rapidly changing absorption of the pump which is expected to vary even within the excitation timescale of the pump pulse. To fully describe this effect, we would need to develop a much more complex dynamical model where the rapidly time dependent absorption of the pump is fully accounted for. This is not a trivial task, and is beyond the scope of the current paper.

S4 Two-beam coupling contribution

When two coherent, co-polarised, near-degenerate beams impinge upon a plane, they generate an interference pattern. This gives rise to a spatially dependent change to the index of the material, which can cause diffraction generated signals in the experiment. The description we give below refers specifically to our pump-probe geometry, though it is important to realise that similar coherent signals (i.e. resulting from interference and diffraction) can result in many types of nonlinear optical measurement, even those employing a single focused beam[9].

For the geometry used in Fig. 4c, we model beams in glass (prism) with wavelength 1250 nm wavelength, $\theta_{\text{pr}} = 44.9^\circ$ and $\theta_{\text{pm}} = 48.3^\circ$. These beams will result in the interference pattern inside the ITO, as shown in Figure S5. For the beams used in our experiment ($I_{\text{pm}} = 70\,\text{GW}\,\text{cm}^{-2}$ and $I_{\text{pr}} = 0.13\,\text{GW}\,\text{cm}^{-2}$) we expect the interference pattern shown in Figure S5b, with a spatially dependent oscillation in intensity of $\pm 4\,\text{GW}\,\text{cm}^{-2}$. Due to the intensity dependent index of refraction in ITO, one also expects a spatial dependence to the local index of refraction. Assuming the linear intensity dependence present in Equation S4, one can expect the refractive index of the ITO layer to roughly resemble that shown in Figure S5c. A spatial profile in the index of refraction will act as a diffraction grating, scattering pump light into the direction of the probe beam, and subsequently into our detector. While the spatial modulation in index is relatively small, leading to a relatively weak scattering effect, we only require a small intensity of the much stronger pump beam scattered in the direction of the probe to give a large switching signal. To obtain an estimate of this contribution, we use Comsol to calculate the diffraction pattern expected from the spatially varying index shown in Figure S5c, assuming a 60 nm ITO layer with a uniform refractive index in z direction. This predicts a 1st order diffraction of ~ 0.1 % of the pump beam that will be scattered in the direction of the probe beam. Hence, the signals we measure in our detector are not only based on the zero order reflection of the probe, but also a contribution of the first order

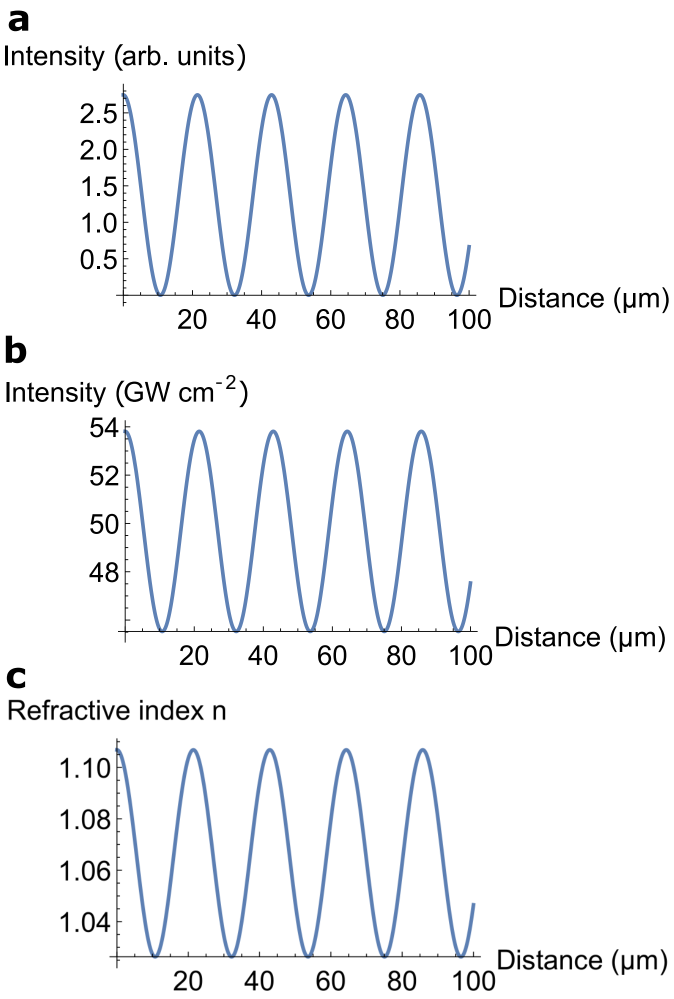

Figure S5. Diffraction through interference induced refractive index grating. a, Interference pattern of two equally intense beams (44.9°, 1250 nm) and (48.3°, 1250 nm) in glass. **b**, Interference pattern of 70 GW/cm^2 pump (44.9°, 1250 nm) and 0.13 GW cm^{-2} probe (48.3°, 1250 nm) at the glass/ITO interface. **c**, Corresponding refractive index distribution resulting from B, based on Equation S4.

diffraction of the pump. Considering the spatial overlap required for the pump to be scattered, we can directly estimate the contribution of scattered pump to the "differential reflection" signal: this corresponds to

$$\Delta R_{\mathrm{CC}} \sim \frac{0.1\ \% * 70\,\mathrm{GW\,cm^{-2}}}{0.13\,\mathrm{GW\,cm^{-2}}} \sim 54\ \% \tag{S21}$$

of the probe intensity. This is even larger than the difference in peak TM and TE experimental signals, which is 15 % (see Fig. 4c). However, due to their different temporal dynamics, the thermal and coherent contributions to the signal are expected to add sub-linearly. Moreover, we note that the refractive index relation used to calculate the scattering of the pump assumes full thermalisation, whereas the ITO will heat up during the evolution of the pump pulse. Thus the 54 % predicted above is an overestimate, and we believe it to be consistent with the measured value of 15 %.

For non-degenerate pump and probe, one expects a non-stationary interference pattern, i.e. one that changes quickly with time. For our geometry, one can easily show that effects of the grating will be washed out within the ∼ 100 fs of our pulses when the pump and probe differ by only a few nm. Again, this is in agreement with our experiments presented in Fig. 4b, which show coherent signal only for near-degenerate measurements.

S5 Probe polarization dependence of the nonlinear effect

In Figure S6 we compare the dynamical model of Fig. 2b with the corresponding TE probe case. The nonlinear optical effects on a TE polarized probe are small, as the shifting resonance feature is only accessible for TM polarized light (see e.g. in

[Figure S1](#)). However, these small nonlinear changes to the TE reflection/absorption are beneficial as changes to the TE pump absorption are negligible, enabling easier modelling of time dependent effects.

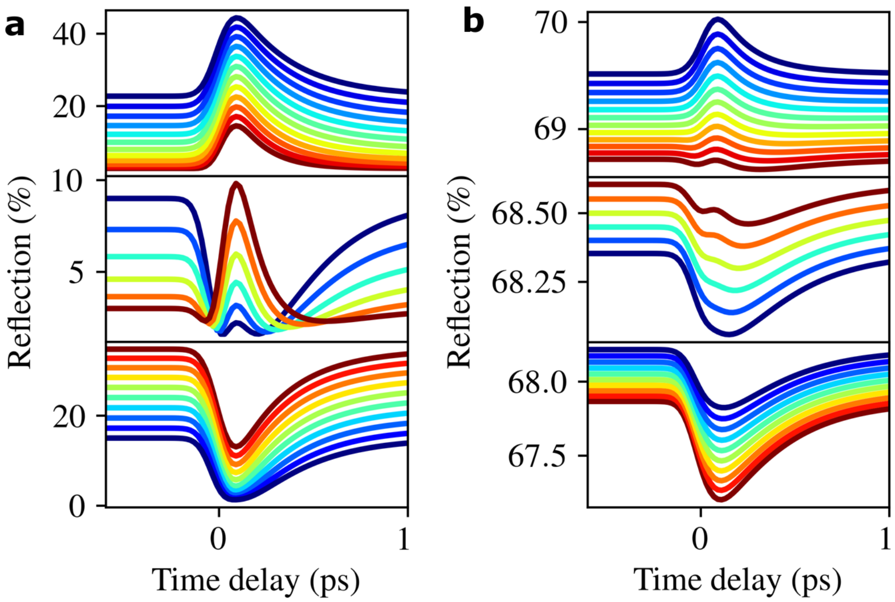

Figure S6. Polarization dependent nonlinear reflection. a, The TM probe case as seen in Fig. 2b or Figure S2 (pr: TM, 48.3°, $f_{pr} = 261\dots207$ THz; pm: TE, $f_{pm} = f_{pr}$). **b**, The same case except for the probe being TE polarized, leading to only small reflection changes of $\Delta R < 1$ %. (pr: TE, 48.3°, $f_{pr} = 261\dots207$ THz; pm: TE, $f_{pm} = f_{pr}$)

S6 TE-TE measurement

Due to the weak nonlinear response of the TE probe we expect only small thermal contributions to nonlinear measurements as discussed in section S5. However, if the pump is also TE polarised one expects a notable two-beam coupling contribution as the refractive index modulation inside the ITO layer remains similar to the case discussed in section S4. This is indeed what we observe in the TE-TE experiment as seen in Figure S7. The resulting TBC can be seen as a 10% relative reflection increase when near degeneracy of pump and probe, and is significantly larger than the thermal effect in the same measurement.

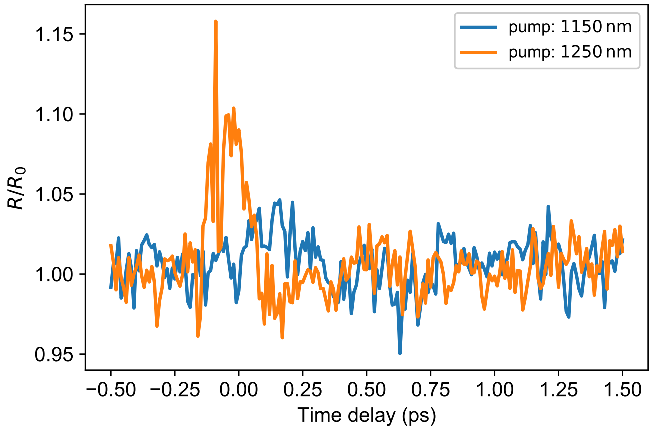

Figure S7. TE-TE measurement. The absence of the thermal response for a TE probe leads to an isolated clear TBC feature when pumping with a TE polarized beam. (pr: TE, 45°, $f_{pr} = 240$ THz; pm: TE, $f_{pm} = 240, 260$ THz)

S7 Coverslip measurement

We have performed measurements at 45°, both beams TM polarized and both wavelength set to 1200 nm (see Figure S8). We first insert the ITO sample, align and measure the time delay scan (1). After that we exchange the sample for a cover slip and only adjust the tilt of the sample holder and the angle of the sample holder to maximize reflection and compensate changes due to slight changes in index matching fluid binding to the prism. Then we measure time delay scan (2). Finally, we change back to ITO, again, only adjusting tilt and angle to retrieve the maximum reflection (for 1500 nm, due to higher reflection). With the then measured scan (3) we show that the nonlinear response was perfectly reproduced. Importantly, the response of the coverslip alone (2) is considerably smaller.

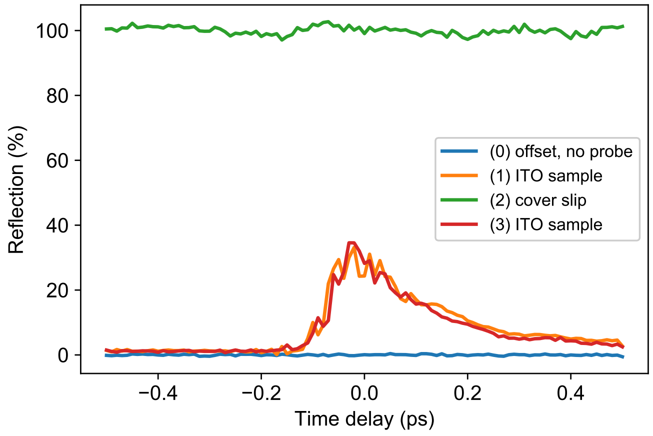

Figure S8. Coverslip measurement. To show the negligible nonlinear response of the prism, index matching fluid and prism we present a delay scan without ITO layer (2). To ensure the alignment not being influenced we take ITO measurements before and after with the same alignment procedure (1,3). We also show the electronic offset without a probe, corresponding to $R = 0$ %. (pr: TM, 45°, $f_{\text{pr}} = 250$ THz; pm: TM, $f_{\text{pm}} = 250$ THz)

We have checked the absence of signal for case (2) for other angles, and signals are always considerably smaller than when the ITO is present, hence we can unequivocally attribute the bulk of the signal to the ITO film.

S8 Autocorrelation measurement

The autocorrelation of our pulses at 1200 nm was measured using an APE pulseCheck autocorrelator (see Figure S9). The measured pulse length is 107 ± 5 fs, with no significant change in pulse length over the range in wavelengths employed in this work. The measurement does not indicate any chirp like behaviour.

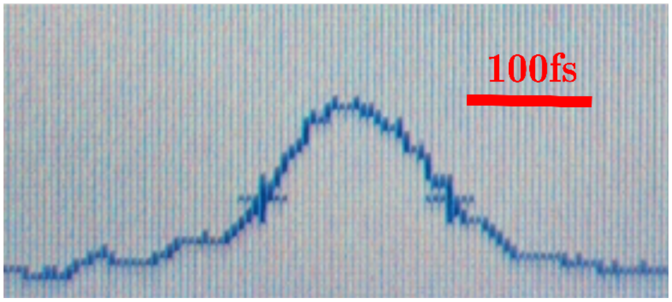

Figure S9. Autocorrelation for pulse length and quality check. The autocorrelation was measured after the pulses passed through the prism with a wavelength of 1200 nm. The pulse has been measured multiple times, leading to a pulse time estimate of 107 ± 5 fs.

References

1. Yang, F., Sambles, J. R. & Bradberry, G. W. Long-range surface modes supported by thin films. *Phys. Rev. B* **44**, 5855–5872, DOI: 10.1103/PhysRevB.44.5855 (1991).

2. Alam, M. Z., De Leon, I. & Boyd, R. W. Large optical nonlinearity of indium tin oxide in its epsilon-near-zero region. *Science* **352**, 795–797, DOI: 10.1126/science.aae0330 (2016).

3. Yang, Y. *et al.* Femtosecond optical polarization switching using a cadmium oxide-based perfect absorber. *Nat. Photonics* **11**, 390–395, DOI: 10.1038/nphoton.2017.64 (2017).

4. Scalora, M. *et al.* Electrodynamics of conductive oxides: Intensity-dependent anisotropy, reconstruction of the effective dielectric constant, and harmonic generation. *Phys. Rev. A* **101**, 053828, DOI: 10.1103/PhysRevA.101.053828 (2020).

5. Secondo, R., Khurgin, J. & Kinsey, N. Absorptive loss and band non-parabolicity as a physical origin of large nonlinearity in epsilon-near-zero materials. *Opt. Mater. Express* **10**, 1545, DOI: 10.1364/ome.394111 (2020).

6. Byrnes, S. J. Multilayer optical calculations. *Prepr. at http://arXiv.org/quant-ph/0208066* (2016).

7. Chambers, L. G., Bender, C. M. & Orszag, S. A. Advanced Mathematical Methods for Scientists and Engineers. *The Math. Gazette* **63**, 139, DOI: 10.2307/3616036 (1979).

8. Kinsey, N. *et al.* Epsilon-near-zero Al-doped ZnO for ultrafast switching at telecom wavelengths. *Optica* **2**, 616, DOI: 10.1364/OPTICA.2.000616 (2015).

9. Yao, B., Ren, L. & Hou, X. Z-scan theory based on a diffraction model. *J. Opt. Soc. Am. B* **20**, 1290, DOI: 10.1364/josab.20.001290 (2003).
